# Correction: Multi-Level Interactions between the Nuclear Receptor TRα1 and the WNT Effectors β-Catenin/Tcf4 in the Intestinal Epithelium

**DOI:** 10.1371/journal.pone.0093418

**Published:** 2014-03-20

**Authors:** The PLOS ONE Staff

In sub-table S1A of Table S1, the nucleotide sequence of Tcf7l2 (Tcf4) should read: “F: CACAGCTCAAAGCATCAGGA R: CTGCATGTGAAGCTGTCGTT.” The length of the amplicon should be indicated as: “242bp.”
